# Diffusion tensor cardiovascular magnetic resonance in hypertrophic cardiomyopathy: a comparison of motion-compensated spin echo and stimulated echo techniques

**DOI:** 10.1007/s10334-019-00799-3

**Published:** 2019-11-22

**Authors:** Zohya Khalique, Andrew D. Scott, Pedro F. Ferreira, Sonia Nielles-Vallespin, David N. Firmin, Dudley J. Pennell

**Affiliations:** 1 Cardiovascular Magnetic Resonance Unit, Royal Brompton Hospital, Sydney Street, London, SW3 6NP UK; 2 National Heart and Lung Institute, Imperial College, London, SW7 2AZ UK

**Keywords:** Diffusion tensor, Mean diffusivity, Fractional anisotropy, E2A mobility, Hypertrophic cardiomyopathy

## Abstract

**Objectives:**

Diffusion tensor cardiovascular magnetic resonance (DT-CMR) interrogates myocardial microstructure. Two frequently used in vivo DT-CMR techniques are motion-compensated spin echo (M2-SE) and stimulated echo acquisition mode (STEAM). Whilst M2-SE is strain-insensitive and signal to noise ratio efficient, STEAM has a longer diffusion time and motion compensation is unnecessary. Here we compare STEAM and M2-SE DT-CMR in patients.

**Materials and methods:**

Biphasic DT-CMR using STEAM and M2-SE, late gadolinium imaging and pre/post gadolinium T1-mapping were performed in a mid-ventricular short-axis slice, in ten hypertrophic cardiomyopathy (HCM) patients at 3 T.

**Results:**

Adequate quality data were obtained from all STEAM, but only 7/10 (systole) and 4/10 (diastole) M2-SE acquisitions. Compared with STEAM, M2-SE yielded higher systolic mean diffusivity (MD) (*p* = 0.02) and lower fractional anisotropy (FA) (*p* = 0.02, systole). Compared with segments with neither hypertrophy nor late gadolinium, segments with both had lower systolic FA using M2-SE (*p* = 0.02) and trend toward higher MD (*p* = 0.1). The negative correlation between FA and extracellular volume fraction was stronger with STEAM than M2-SE (*r*^2^ = 0.29, *p* < 0.001 STEAM vs. *r*^2^ = 0.10, *p* = 0.003 M2-SE).

**Discussion:**

In HCM, only STEAM reliably assesses biphasic myocardial microstructure. Higher MD and lower FA from M2-SE reflect the shorter diffusion times. Further work will relate DT-CMR parameters and microstructural changes in disease.

**Electronic supplementary material:**

The online version of this article (10.1007/s10334-019-00799-3) contains supplementary material, which is available to authorized users.

## Introduction

Diffusion tensor (DT) cardiovascular magnetic resonance (CMR) is used to obtain non-invasive measures of myocardial microstructure [1–3]. Parameters such as mean diffusivity (MD) and fractional anisotropy (FA) describe the freedom of water motion, and degree of myocardial microstructural organisation. Helix angle (HA) and second eigenvector angulation (E2A) relate to the orientation of cardiomyocytes and sheetlets, respectively. Cardiomyocytes take a left-handed helical arrangement at the epicardium and progress smoothly through a circumferential orientation at the mesocardium to a right-handed helical arrangement at the endocardium. This microarchitecture is generally preserved through the cardiac cycle [4]. Sheetlets are dynamic bundles of myocytes that align near wall-perpendicular in diastole and re-orientate to become more wall-parallel in systole, thus effecting wall thickening [4–6]. E2A is a measure of sheetlet alignment and the change in E2A between peak systole and diastole is known as sheetlet mobility [4].

**Fig. 1:**
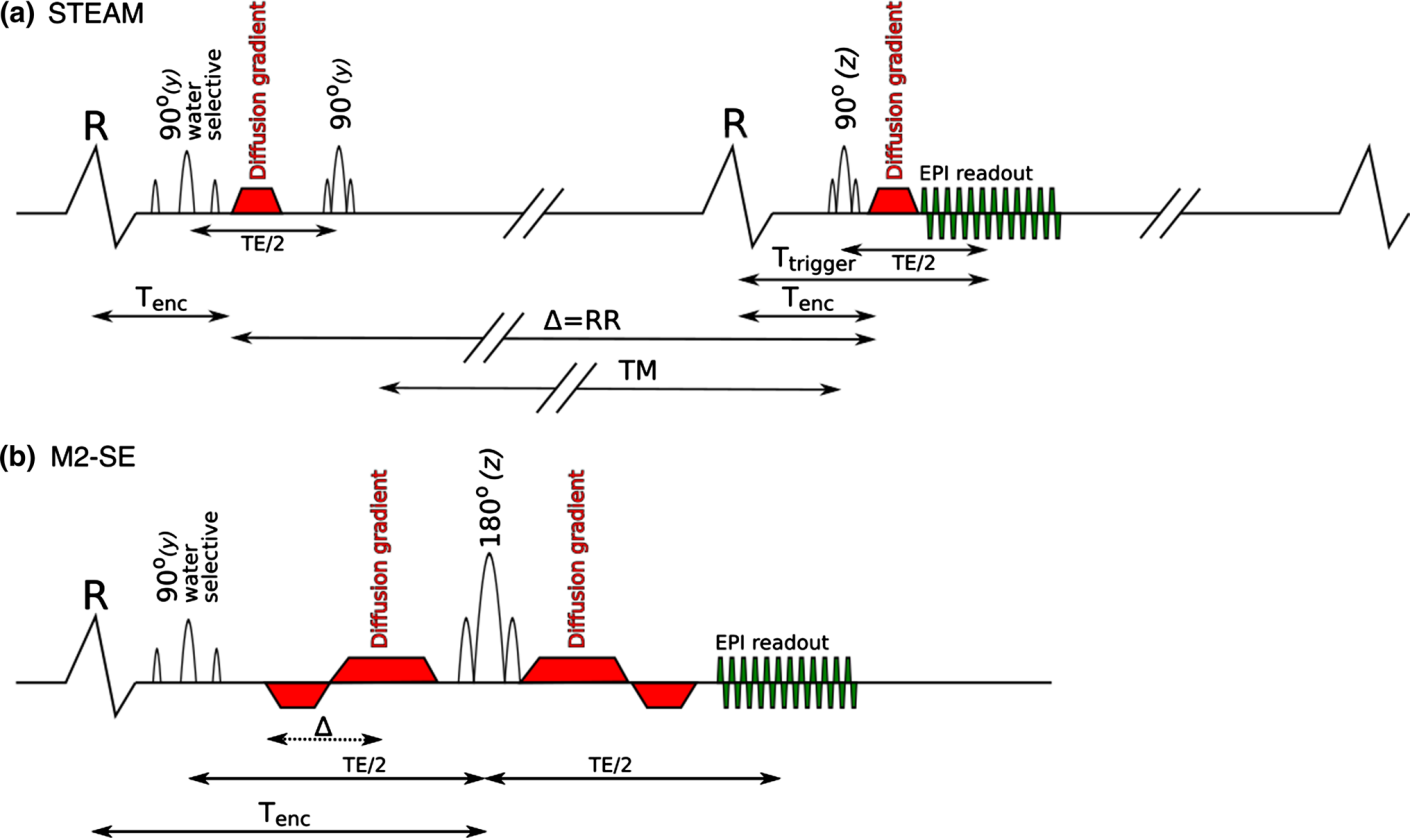
Schematic diagrams of the STEAM and M2-SE sequences used. The key difference between the two sequence times is the period over which the measured diffusion takes place, shown by ∆. The STEAM sequence (**a**) runs over 2 cardiac cycles, whilst the M2-SE sequence (**b**) is triggered to every R-wave. ∆ is shown as a dotted line in b because, unlike the STEAM sequence, the asymmetric gradient design means that the diffusion time is not uniquely defined for the M2-SE sequence. The letters above the RF pulses indicate the axis on which the corresponding slice selective gradient is played out. *T*_*enc*_ time from R-wave to effective time of diffusion encoding, *TM* mixing time, *RR* RR-interval, *T*_*trigger*_ time from R-wave to central k-space line, Δ the diffusion time of the sequence. Note, that this is a schematic diagram and timings are not shown to scale. Modified from Scott et al. [11]

There are currently two main DT-CMR sequences employed in the in vivo assessment of the myocardium (Fig. 1); STimulated Echo Acquisition Mode (STEAM) and second-order motion-compensated spin echo (M2-SE). STEAM is the more established technique, having been used to characterise both healthy cohorts and also patients with cardiac pathology including cardiomyopathy [2, 4, 7–9]. In hypertrophic cardiomyopathy (HCM), STEAM DT-CMR has shown elevated diastolic E2A, which results in reduced E2A mobility [7] and that reduction of FA in hypertrophied myocardium is associated with ventricular arrhythmia and possibly myocyte disarray [9].

However, STEAM suffers from an intrinsically low signal–noise-ratio (SNR), the limited potential confounding effects of strain [1, 10], as well as dependence upon a regular heart rate. Alternatively, M2-SE could offer benefits; it is strain-insensitive, has a higher SNR and has shown promising results in healthy volunteers scanned using high-performance gradient systems at 1.5 T during systole [3, 11]. In recent work, we compared STEAM and M2-SE at multiple cardiac phases in healthy volunteers at 3 T using a widely available gradient specification [12]. However, while a limited number of studies in disease have used a related diffusion-prepared technique for diffusion-weighted and, more recently, diffusion tensor imaging with limited success [13, 14], M2-SE DT-CMR has only been used in patients in one recent study [15] and has yet to be compared to STEAM in a patient cohort.

This study compares STEAM and M2-SE DT-CMR techniques in HCM patients. It aims to establish technique viability, compare diffusion parameters from both sequence types and better understand the relative value of the different approaches to characterise HCM.

## Materials and methods

This study was approved by the National Research Ethics Committee and all participants provided informed written consent. HCM patients were prospectively recruited. The diagnosis of HCM followed the 2011 ACCF/AHA Guideline for the Diagnosis and Treatment of Hypertrophic Cardiomyopathy and the 2014 ESC Guidelines on diagnosis and management of hypertrophic cardiomyopathy criteria [16, 17]. Patients who had undergone previous alcohol ablation or septal myectomy were excluded.

### Image acquisition

CMR was performed on a 3 T scanner (Magnetom Skyra, Siemens, Erlangen, Germany), with an 18-element anterior matrix coil and 8–12 elements of a matrix spine coil. The scanner has a maximum gradient amplitude of 43mT/m per axis and a maximum slew rate of 200 T/m/s. Images were acquired as follows: bSSFP, DT-CMR, T1 mapping, LGE, post-contrast T1 (see additional material for flowchart).

### bSSFP images

Retro-gated balanced steady-state free precession (bSSFP) cine images were acquired in the 3 long-axis planes and a stack of short-axis slices running from the atrioventricular ring to include the apex. These were used for volumetric analysis and assessment of maximum wall thickness. They were also used to identify a suitable mid-ventricular slice, which included an area of left ventricular hypertrophy (LVH) for subsequent DT-CMR imaging. Using the cine data, the timing of end systole and diastole was identified in the selected slice.

### DT-CMR acquisition

DT-CMR was performed with STEAM and M2-SE in systole and then diastole. To avoid breath-holding fatigue affecting image quality, the order of STEAM and SE acquisitions was alternated for consecutive patients. Imaging was performed at the end-systolic and late diastole time points. The DT-CMR slice position was tracked between the systolic and diastolic acquisitions using linear tagged images acquired in perpendicular long-axis planes [7].

The STEAM and M2-SE DT-CMR sequences were implemented as in our prior healthy volunteer comparison [12] and are shown schematically in Fig. 1.

Imaging parameters were matched between sequences where possible and an identical EPI readout was used in both cases. EPI readout duration was reduced by a “zone-selected” technique [18] and a SENSE [19] acceleration factor of 2. Acquired in plane spatial resolution was 2.8 × 2.8 mm^2^, reconstructed to 1.4 × 1.4 mm^2^ and slice thickness was 8 mm. All flip angles were 90° for the STEAM sequence and flip angles were 90° and 180° for the M2-SE sequence. The final RF pulse in each sequence (the 3rd 90° pulse for STEAM and 180° pulse for M2-SE) was matched in bandwidth-time product (6) and duration (3.3 ms) and the full-width half-maximum of the slice profiles was matched between sequences. Each image is acquired in 1 cardiac cycle for the M2-SE sequence, while the STEAM sequence acquires one image every other cardiac cycle. Each STEAM breath hold is 18 RR-intervals and each M2-SE breath hold is 16-RR intervals. The first 4 (STEAM) or 2 (M2-SE) RR-intervals in each breath hold are used for acquiring EPI phase correction lines and parallel imaging reference lines. The next 2 (STEAM) or 1 (M2-SE) cardiac cycles are used to obtain the “b0” image, followed by 2 (STEAM) or 1 (M2-SE) cardiac cycle for each of the 6 diffusion encoding directions used. For the M2-SE sequence the “b0” and 6 diffusion-encoding directions are acquired a second time to provide an additional signal average. A maximum of 16 breath-holds were acquired with a diffusion encoding of *b*_main_ = 450 s mm^−2^, corresponding to 8 averages of the STEAM and 16 averages of the M2-SE data. An additional breath hold was acquired for both sequences with *b*_ref_ = 150 s mm^−2^ to minimise myocardial blood signal in M2-SE and the effects of microvascular perfusion on the reconstructed tensors [12]. Water-selective excitation was used for fat suppression in both sequences. TE was 25 ms for STEAM and 76 ms for M2-SE.

M2-SE DT-CMR acquisitions were timed to place the echo at peak systole or during the most stationary phase of diastole based on the bSSFP cine data in the same imaging plane. The timing of the STEAM acquisitions was adjusted to ensure the centre of the diffusion encoding was matched between sequences.

### T1 mapping

Myocardial T1 mapping was performed using a Modified Look Locker Imaging (MOLLI) sequence [20] with a bSSFP readout. Before contrast administration, a 5(3)3 protocol with a minimum inversion time of 112 ms and steps of 80 ms was used. Acquired spatial resolution was 1.4 × 2.1 mm^2^ (readout × phase) at a field of view of 360 × 307 mm^2^ and a slice thickness of 8 mm. TE = 1.1 ms, TR = 2.7 ms and flip angle was 20°. 7/8ths partial Fourier and GRAPPA acceleration factor 2 were used. The acquisition was repeated to provide two averages.

Post-contrast T1-mapping was performed after the late gadolinium enhancement images for use in calculating extracellular volume (ECV) fraction estimates using a 4(3)1(1)2 protocol with minimum inversion time 100 ms and 80 ms steps. Acquired spatial resolution was 1.9 × 2.4 mm^2^ with the same field of view, slice thickness, number of averages and acceleration methods as in the pre-contrast acquisition. TE = 1.0 ms, TR = 2.4 ms and flip angle 20°.

### Late gadolinium enhancement imaging

Gadobutrol injection (Gadovist 1.0 mmol/ml solution at 0.1 mmol/kg) was used for contrast. Late gadolinium enhancement (LGE) images were acquired using a standard phase-sensitive inversion recovery-spoiled gradient echo at a spatial resolution of 1.5 × 1.5 mm^2^ and a slice thickness of 8 mm. Inversion time was adjusted to null the “normal” myocardium and the planes imaged included a short-axis stack with a gap between slice edges of 2 mm.

### CMR image analysis

Volumetric analysis was performed using CMRtools (Cardiovascular Imaging Solutions, London, UK). Volumes and mass were indexed to body surface area [21]. Images were reviewed to classify areas of LV hypertrophy and late gadolinium enhancement (LGE) in a 4-segment model for initial assessment and then a clockwise 12-segment model by the consensus of two operators; segment 1 encompassed the superior insertion point. The latter model was used to assess correlations with DT-CMR results. LGE quantification was performed using CMR42 software (Circle Cardiovascular Imaging, Calgary, Canada). The full-width half-maximum technique was used to quantify enhancement and expressed as a percentage of LV mass (excluding papillary muscles) for the DT-CMR-matched slice.

### DT-CMR analysis

DT-CMR data were processed using MATLAB (Mathworks, Natick, MA, USA) software as described previously [7]. Only *b*_ref_ = 150 s mm^−2^ and *b*_main_ = 450 s mm^−2^ images were used (without averaging) in the tensor calculation and *b* values for the STEAM acquisitions were corrected for the heart rate. Images with visually apparent motion-related signal loss were manually excluded from the tensor calculation and the remaining images were registered using a rigid translation. Pixels containing blood with high signal intensity in the M2-SE acquisitions were nulled based on their intensity prior to the image registration step and the original signal intensities were returned after registration. Maps of helix angle (HA), absolute value of the secondary eigenvector angle (E2A), fractional anisotropy (FA), mean diffusivity (MD) were generated [7]. Wall thickness-normalised helix angle gradient (HAG) in ^o^/% was calculated from epi- to endocardium radial profiles [2, 22]. Average parameters (median for E2A, mean for others) were calculated for the whole LV within the slice and by the same 12-segment model used for LGE and LVH analysis. Mean LV signal-to-noise ratio (SNR) for the *b* = 450 s/mm^2^ data were estimated via the multiple-repetitions method, described previously [23, 24]. The mean was calculated over all diffusion directions and the median over subjects.

Quality of DT-CMR data was assessed using a scoring system of HA maps [12]. Scores of 0 for < 50%, 1 for > 50%, 2 for > 75%, and 3 for > 95% normal transmural HA variation were allocated. Maps with scores of 0 were excluded from further analysis. Scoring was performed separately in a randomised order by two blinded observers (5 and 3 years of DT-CMR experience) and conflicts were resolved by consensus.

In-house software was written in MATLAB to calculate ECV maps based on the pre- and post-contrast T1 maps and haematocrit values measured from a blood sample collected immediately before the scan. T1 maps were registered and the pixelwise ECV was calculated based on Flett et al. [25]. The epi and endocardial borders were manually defined and the superior insertion point was selected. The tool calculated 12-segment ECV based on the same model used for the DT-CMR, LGE and LVH analysis.

DT-CMR findings were also assessed by areas with both hypertrophy and LGE (LVH + LGE + ) and those with neither (LVH − LGE − ). Statistical comparison of the regional findings by both techniques was performed in systole.

One problem with the M2-SE acquisitions in diastasis, that was also highlighted in our previous work in healthy volunteers [12], is frequent missed-triggers. Based on the time stamps on the DICOM files and an expected range of RR-intervals (500–1500 ms), we estimated the proportion of images that were acquired with an incorrectly detected or missed R-wave.

### Statistical analysis

Summary statistics were presented as median and interquartile range [IQR]. The Wilcoxon-signed-rank-test was used to compare STEAM and M2-SE values and Pearson’s correlation with linear regressions were used to assess correlations. Statistical analysis was undertaken using MATLAB with the Statistics and Machine Learning Toolbox.

### Phantom imaging

One difference between the two DT-CMR methods not described in previous work is the differing influence of eddy currents on the results. In this work, we performed DT-CMR acquisitions in a structured phantom to assess the degree of eddy current-related artefacts. The phantom was constructed from a 145 mm inner diameter cylindrical container filled with agar (40 g agar per litre of tap water, see [11] for approximate T1, T2 and MD values). Geometric structure was created using a toy construction kit (Lego). Rods spaced 31 mm apart in a grid-like formation were fixed parallel to the axis of the cylindrical container. The phantom was placed with the cylinder axis vertically and imaging was performed using the STEAM and M2-SE DT-CMR techniques described above. For reference, a turbo-spin echo acquisition was also performed in the same plane with a 200 × 200 mm field of view, 256 × 256 acquired matrix, 5 mm slice thickness, 12 ms TE, 2000 ms TR, echo train length of 5, 130 Hz/pixel bandwidth and flip angles of 90° and 180°. The imaging plane was selected to show the cylindrical phantom in cross-section (a coronal slice).

## Results

The baseline characteristics of the patients are shown in Table 1. All patients had asymmetrical septal hypertrophy and presence of LGE. Within the slice imaged with DT-CMR, all patients had areas of hypertrophy and LGE in the septum but not in the lateral wall.

**Table 1:** Baseline characteristics of the hypertrophic cardiomyopathy patients

	HCM patients *n* = 10
Age (years)	64 [11]
Male	7 (70%)
Body surface area (m^2^)	2.03 [0.4]
Heart rate (beats/minute)	63 [8]
Whole heart characteristics	
Asymmetric septal hypertrophy	10 (100%)
Late gadolinium enhancement Anterior Septal Inferior Lateral	4 (40%) 10 (100%) 3 (30%) 1 (10%)
Indexed LV EDV (mL/m^2^)	70 [16]
Indexed LV ESV (mL/m^2^)	21 [10]
LV ejection fraction (%)	76 [3]
LV mass index (g/m^2^)	92 [39]
Maximum end-diastolic wall thickness (mm)	20 [5]
Slice characteristics	
Hypertrophy location Anterior Septal Inferior Lateral	2 (20%) 10 (100%) 2 (20%) 0 (0%)
Late gadolinium enhancement Anterior Septal Inferior Lateral	5 (50%) 10 (100%) 1 (10%) 0 (0%)
Late gadolinium enhancement (g)	1.5 [2.9]
Late gadolinium enhancement (% slice mass)	5 [13]
Extracellular volume fraction (%)	26 [2]

Median [IQR] or number of patients (%)

*LV* left ventricle, *EDV* end-diastolic volume, *ESV* end-systolic volume

**Fig. 2:**
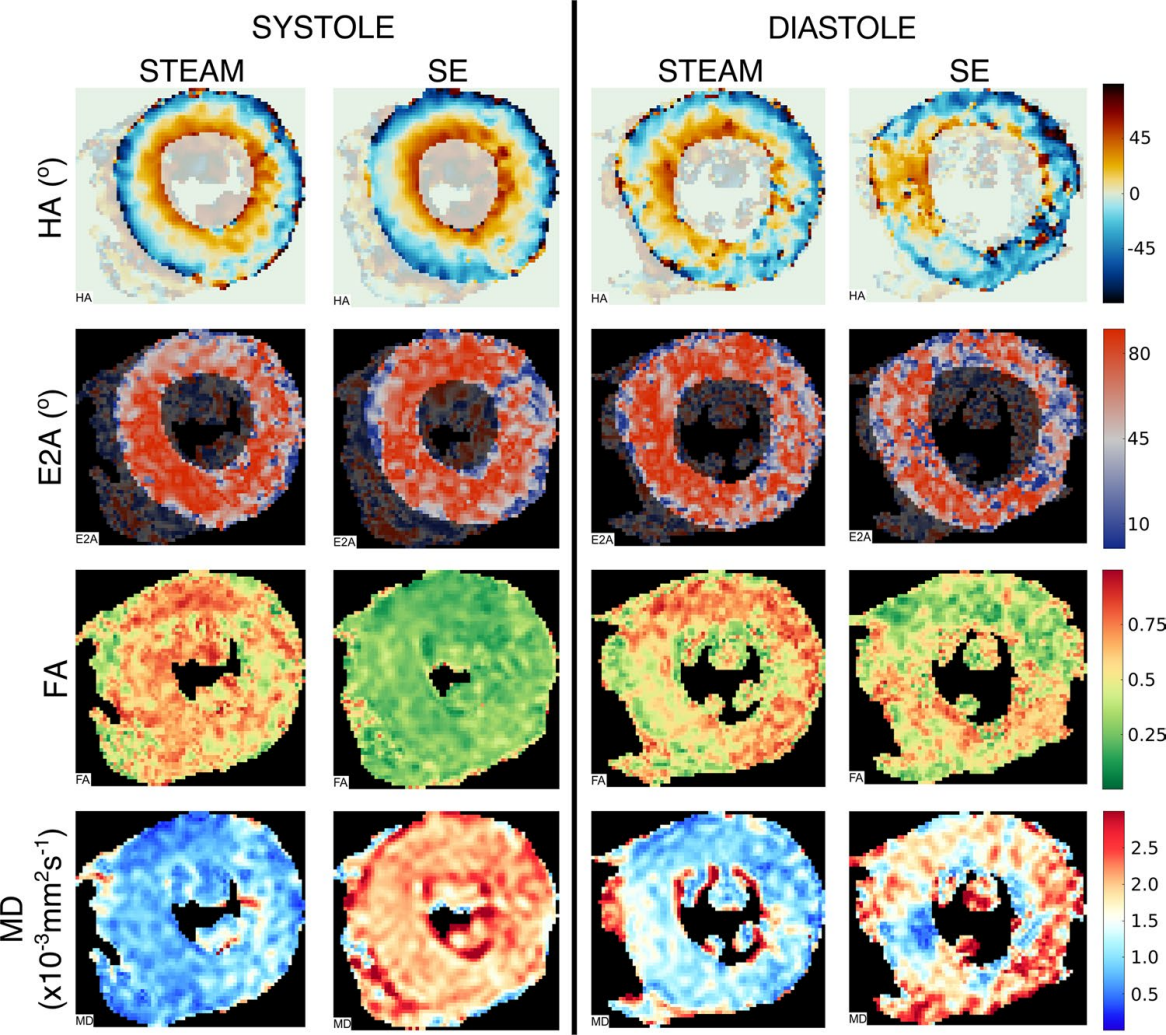
Example DT-CMR results maps obtained in an HCM subject with both sequences at both cardiac phases. Systolic data from both sequences were of sufficient quality for analysis, while only the STEAM sequence was of sufficient quality in diastole. The diastolic M2-SE data were rejected as > 50% of the helix angle map deviated from the expected pattern in the myocardium

All patients completed the scan protocol. Typical scan duration was 90 min. Median [IQR] breath-holds in diastole were 11 [4] for STEAM and 12 [7] for M2-SE (*p* = 0.20) and in systole 11 [2] in STEAM and 11 [3] for M2-SE and (*p* = 0.53). Median [IQR] scores for quality of HA maps were STEAM diastole: 2 [2], M2-SE diastole: 0 [1], STEAM systole: 2 [2], M2-SE systole: 1 [2]. Data were analysable from all STEAM acquisitions, but only 7/10 (systole) and 4/10 (diastole) M2-SE acquisitions. Typical examples of DT-CMR parameter maps are shown in Fig. 2.

Missed-triggers occurred in 0 STEAM systole, 1 M2-SE systole, 3 STEAM diastole and 7 M2-SE diastole acquisitions. For the STEAM data and M2-SE systole data, we estimate that there was a maximum missed-trigger rate of 2.5%. In contrast, for the M2-SE diastole data there were acquisitions with 0.7, 1, 5, 7, 12, 18 and 19% missed-trigger rates. Of the acquisitions with missed-triggers rates > 10%, 2 of the 3 were rejected due to poor quality data. Median [IQR] SNR for data sets scoring > 0 was 7.4 [1.9], STEAM diastole, 4.2 [1.3] M2-SE diastole, 8.5 [1.6] STEAM systole and 5.6 [1.9] M2-SE diastole.

**Fig. 3:**
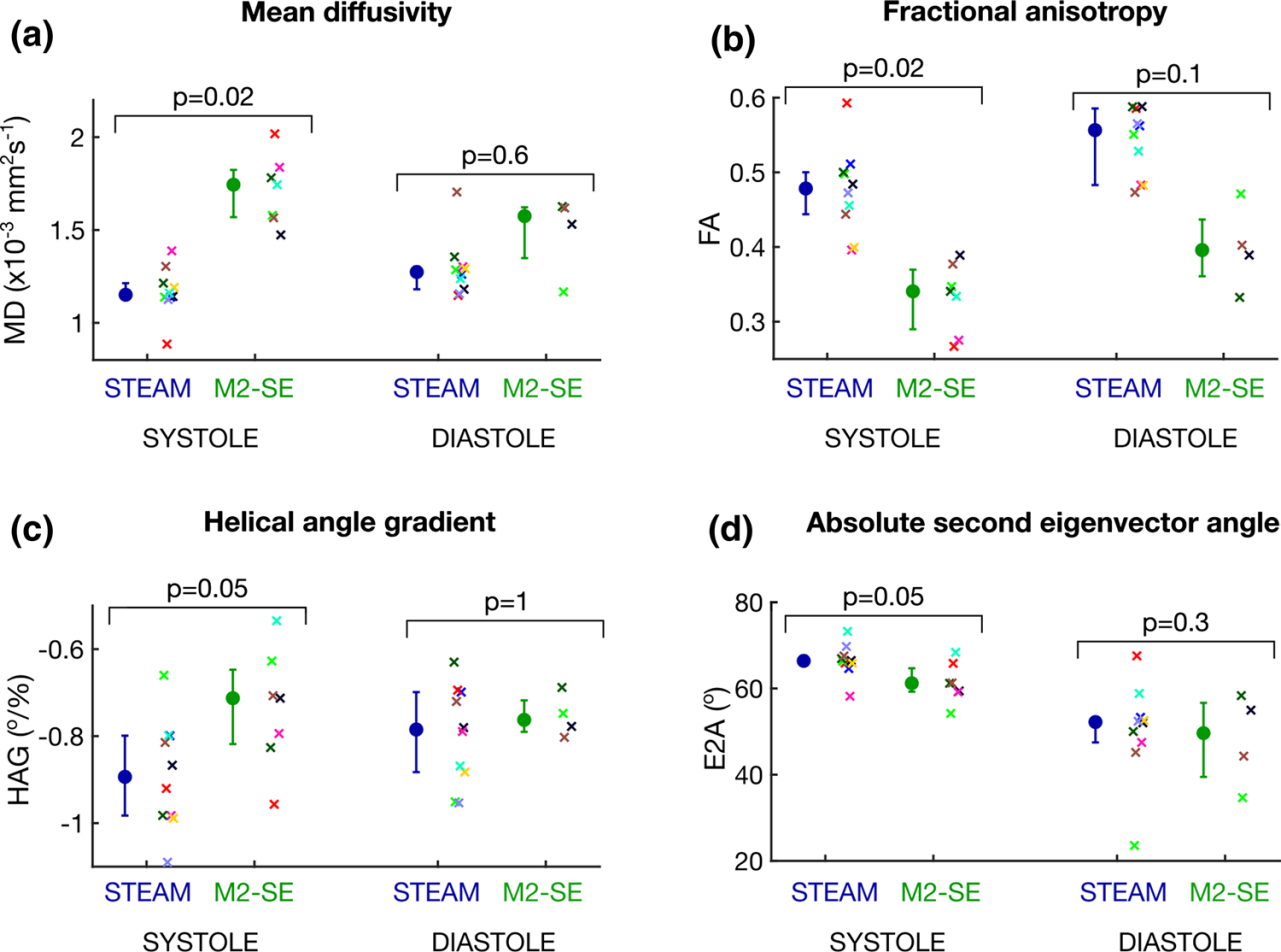
Plots of global DT-CMR results compared between sequences at both cardiac phases: **a** mean diffusivity, **b** fractional anisotropy, **c** helix angle gradient and **d** absolute second eigenvector angles. Mean diffusivity was higher and fractional anisotropy lower when measured by M2-SE. Helix angle gradient was steeper using STEAM. Both techniques had comparable findings for E2A in diastole

Figure 3 and Supplementary Table 1 show that the average myocardial DT-CMR results over the mid short-axis slice varied by sequence at both cardiac phases. For MD, M2-SE yielded higher values at both phases (*p* = 0.02 and *p* = 0.6 in systole and diastole, respectively). Conversely, FA was lower when obtained using M2-SE at both phases (*p* = 0.02 and *p* = 0.1). Biphasic HAG was steeper and E2A was higher when measured using STEAM at both phases, though not significantly.

**Fig. 4:**
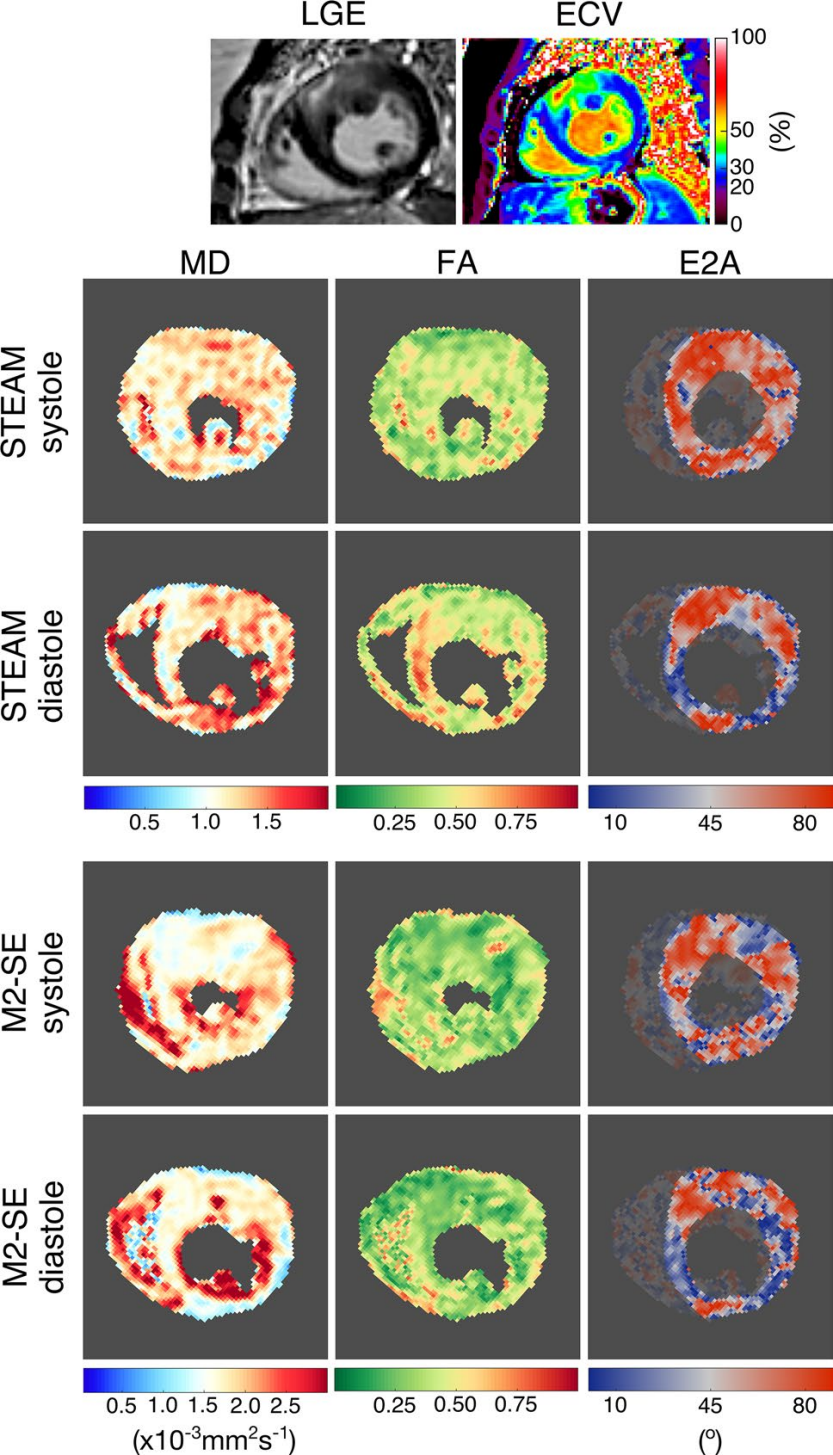
Example DT-CMR maps for both sequence types with corresponding late gadolinium image and extracellular volume fraction map. Mean diffusivity, fractional anisotropy and E2A maps are shown. The scale on the MD map is modified to account for the higher values found with M2-SE

**Fig. 5:**
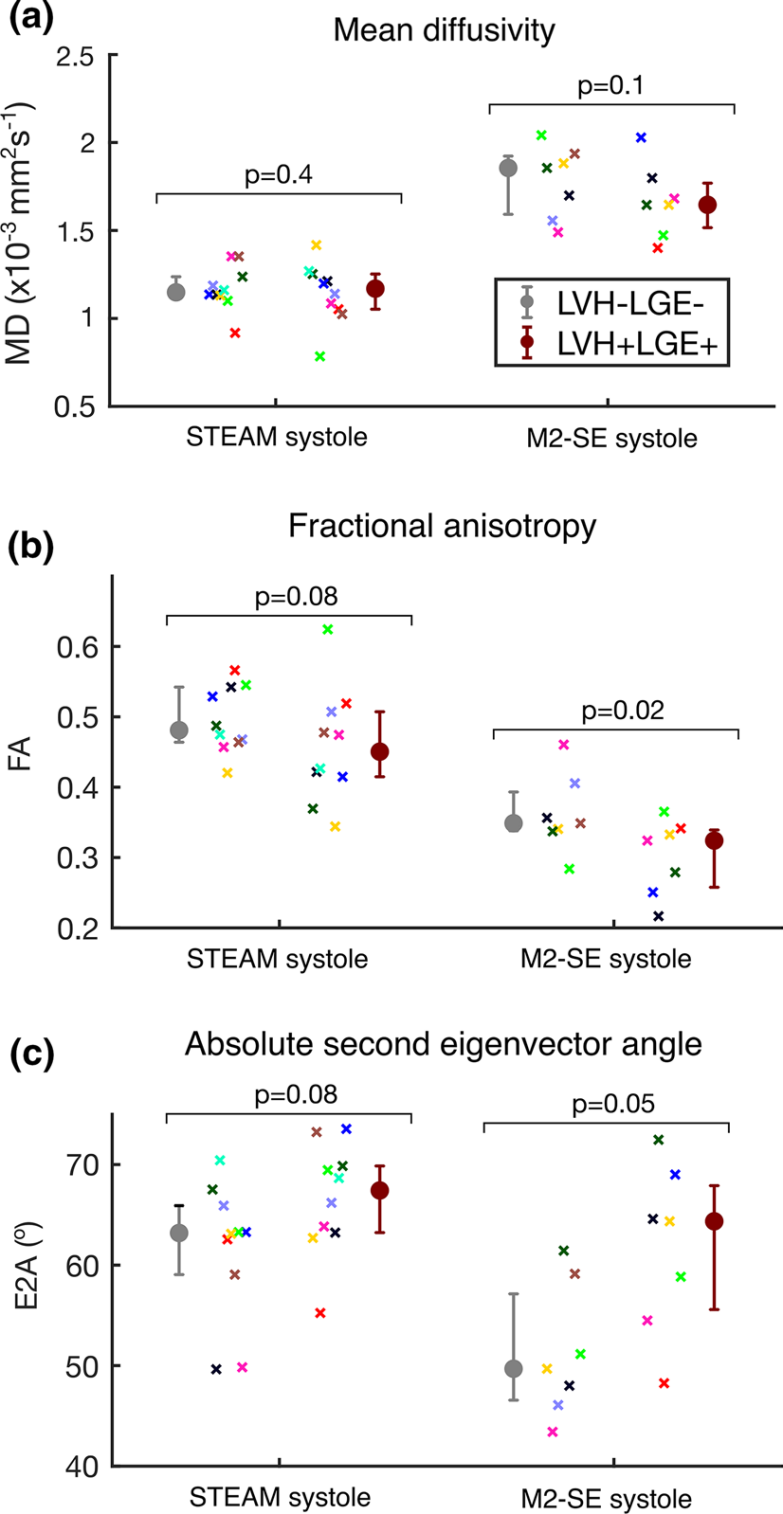
DT-CMR findings segregated by areas with and without both hypertrophy and fibrosis (LVH + LGE + vs LVH − LGE − ). Plots show differences in **a** mean diffusivity, **b** fractional anisotropy and **c** absolute second eigenvector angles

Example DT-CMR maps alongside an LGE image and ECV map are shown in Fig. 4. Results of the 12-segment analysis comparing MD, FA and E2A in LVH + LGE + to LVH − LGE − regions are found in Supplementary Tables 2 and 3 and comparative plots are shown in Fig. 5. Statistical analysis between these regions was only performed in systole due to insufficient data points in diastole and to avoid excessive sub-analysis. Using STEAM, LVH + LGE + areas were distinguished from LVH − LGE − areas by elevated diastolic E2A and reduced E2A mobility (*p* = 0.002). Using M2-SE, FA was significantly lower in LVH + LGE + regions (*p* = 0.02).

**Fig. 6:**
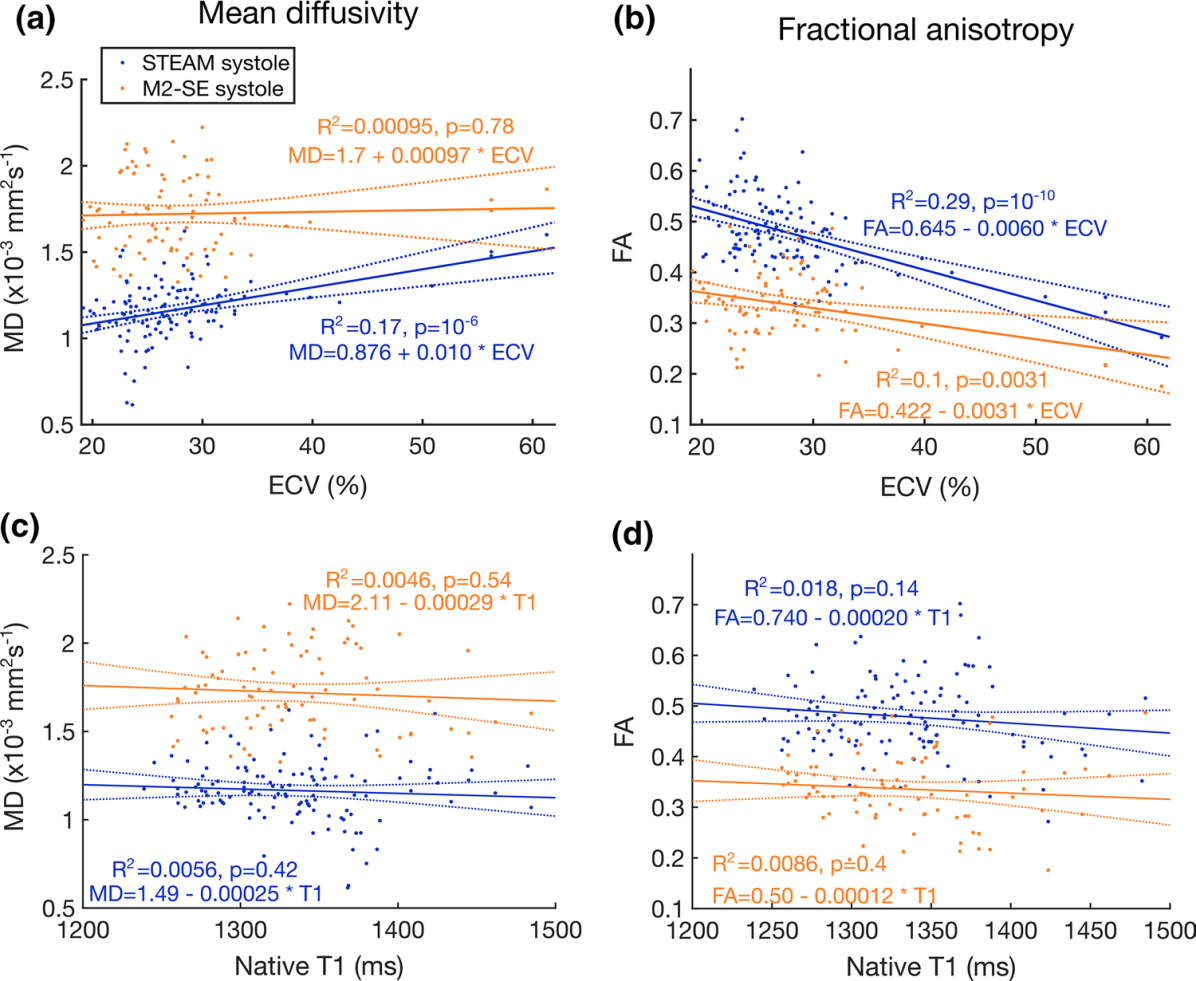
Correlation plots. Mean diffusivity (**a**, **c**) and fractional anisotropy (**b**, **d**) are plotted with extracellular volume (ECV) (**a**, **b**) and native (pre-contrast) T1 (**c**, **d**). Equations of the linear fits are shown on the plots with ECV in % units, MD in × 10^−3^ mm^2 ^s^−1^ and T1 in units of ms (FA is unitless)

Pearson correlations and linear regression were performed between the ECV and native T1 measured in each segment and the MD and FA values in the same segments (Fig. 6). Significant correlations exist between ECV and both MD and FA using STEAM (*R*^2^ = 0.17, *p* < 0.001 and *R*^2^ = 0.29, *p* < 0.001, respectively). Using M2-SE, only FA showed a negative correlation with ECV (*R*^2^ = 0.1, *p* = 0.003). As expected, MD increases and FA reduces with increasing ECV. There were no significant correlations between native T1 and FA or MD measured in systole using either sequence.

### Phantom imaging

**Fig. 7:**
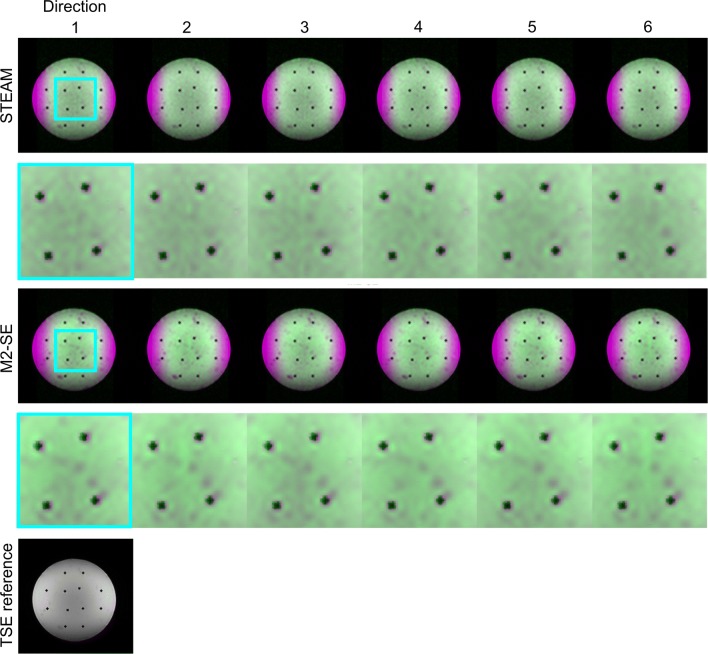
The effect of eddy currents. Phantom images acquired with the STEAM and M2-SE sequence (green) are overlaid on a reference turbo-spin echo sequence (magenta). The differing orientation of the diffusion encoding gradients in each of the six directions results in a different eddy current-related image distortion, which is considerably more evident in the M2-SE data than the STEAM images. The in-plane excitation profile results in lower signal intensity at the edges of the phantom in the phase encode direction in the DT-CMR images. Also see supplementary animation. Note that the internal structure in the phantom is not perfectly aligned as a grid due to the heat of the agar when poured into the container

Figure 7 shows images from the phantom study demonstrating the difference in eddy current artefacts between the two sequences. The same data are also presented as an animation in the supplementary material. While there is little difference in the images between diffusion directions for the STEAM sequence, the effects of eddy currents result in varying geometric distortion between the diffusion encoding directions for the M2-SE sequences.

## Discussion

This study is the first to evaluate and compare two DT-CMR techniques in a patient cohort. We studied patients with HCM as similar DT-CMR sequences [7, 13, 26] have previously identified microstructural abnormalities in these patients independently. We sought to establish and compare the feasibility of both STEAM and M2-SE, provide an initial comparison of DT-CMR results and initial insights into the future clinical and scientific utility of the two methods in patients with HCM.

Results show that currently only STEAM can reliably perform biphasic microstructural assessment in HCM patients. M2-SE is feasible for systolic assessment, but has a high failure rate (60%) in diastole. Our prior work comparing M2-SE and STEAM in healthy volunteers using the same sequences, protocols and scanner found that: 1/15 systolic, 3/15 sweet spot and 7/15 diastolic M2-SE were not evaluable, whereas all but one diastolic STEAM data set were interpretable [12]. While the success rates of M2-SE imaging in both systole and diastole were reduced in the cohort of HCM patients studied here, the reduction relative to our success rates in healthy volunteers is not statistically significant. Based on a binomial distribution, the 95% confidence intervals of the success rates observed in our healthy volunteer study are 68–100% in systole and 27–79% in diastole. This non-significant difference in success rates between healthy volunteers and HCM patients could be a consequence of patient size: (body surface area was substantially larger in HCM subjects, 2.03 [0.4] vs. 1.7 [0.2]); age (HCM subjects were substantially older, 64 [11] vs. 24 [11]); anxiety and experience in the MRI scanner, which is likely to be less and more, respectively; or simply statistical fluctuations. We have also demonstrated the greater influence of eddy current-related artefacts in the M2-SE than in the STEAM acquisitions using a phantom.

DT-CMR data sets were accepted or rejected based on 50% of the myocardium demonstrating the expected progression of HA from a negative or left-handed helical angle at the epicardium, to a positive or right-handed helical arrangement on the endocardium. HA maps were used to assess DT-CMR quality, as HA appears relatively preserved in most pathologies we have studied, thereby avoiding the need to define an arbitrary threshold of some parameter, like MD or SNR, that may also be abnormal as a consequence of disease. Rejected data sets, where < 50% of the myocardium showed a normal appearing HA, tended to have low SNR in the regions of abnormal HA (see Fig. 4 and Supplementary Figure S2 in [12] for examples). The lower success rate of diastolic imaging in both healthy volunteers and HCM patients could relate to the complex cardiac motion during diastole that second-order motion compensation fails to address. However, assessing the magnitude of such motion components would require a technique able to produce accurate and precise measures of tissue displacement or velocity with high temporal resolution, covering the whole cardiac cycle. Neither the DENSE data or feature tracking of the cine data available in this HCM cohort were appropriate for this analysis. In addition, as demonstrated by our analysis of the DICOM timestamps in this work, the longer diffusion gradients (required for motion compensation) extend close to the next R wave and increase the likelihood of missing the next trigger for M2-SE diastolic imaging. This mis-triggering leads to signal loss. We visually inspect all data acquired and aim to reject all acquisitions where there is visible artefact or abnormal signal intensity. However, rejecting images clearly results in a reduction in the diffusion tensor signal-to-noise ratio and a careful balance is required between minimising artefacts and maintaining sufficient data.

Another study demonstrated that diastolic M2-SE DT-CMR is less precise than systolic, with increased uncertainty in all parameters measured in diastole [27]. However, an earlier smaller comparison study of M2-SE and STEAM with sweet-spot imaging at 1.5 T did not reject any data sets and the authors found improved signal to noise ratio when using M2-SE [3].

The maximum gradient strength available on the system used in this work was less than that available in many other M2-SE studies (43 mT/m vs. 80 mT/m) [3]. While eddy current-related artefacts would likely be more severe, higher strength gradients would assist in reducing M2-SE diffusion encoding gradient durations and, therefore the time period over which the sequence is sensitive to higher-order motion-related signal loss.

There was a systematic difference in MD and FA findings between M2-SE and STEAM. This has been reported before both in vivo and ex vivo [3, 12, 28]. The higher MD and lower FA values obtained using M2-SE reflect the shorter diffusion time and, therefore, fewer interactions between water molecules and microstructural barriers as demonstrated in recent simulation work [29]. Whilst these differences between sequences were only statistically significant for systole in this study, the trend was present in diastole and lack of statistical significance may reflect the small numbers in this group. The time-dependent nature of the measured MD and FA means that validation against reference standards is currently not possible for complex biological-like anisotropic media, but validation of HA and E2A as measures of the orientation of cardiomyocytes and sheetlets has been performed in previous studies [4, 30]. The reduction in helix angle gradient using M2-SE relative to STEAM was also previously found in healthy volunteers [12] and could reflect increased noise in the helix angle maps or a consequence of residual blood signal in epi and endocardial pixels in M2-SE data. E2A values were similar between sequences and appear higher in systole than diastole (although statistical comparisons were not performed due to small numbers of diastolic M2-SE data). Interestingly diastolic E2A was similar by both techniques at around 50°, but this value is higher than previously reported values in normals, which are in the region of 15° for STEAM and 30° for M2-SE [3, 7]. The implication is that M2-SE may not be able to clearly detect the diastolic abnormality previously reported in HCM, namely elevated diastolic E2A and failure of diastolic sheetlet relaxation [6, 7].

This study goes further than previous comparisons by examining the ability of both sequences to discriminate areas of LV hypertrophy and fibrosis. However, diastolic findings with M2-SE were not analysed due to the high failure rate and limited number of data points. Both techniques found a lower FA in LVH + LGE + areas, but this was significant only with M2-SE. This FA reduction in phenotypically diseased regions is in accordance with previous studies [9, 26]. STEAM-based studies in HCM have shown that MD was higher and FA values were lower in the septum, possibly due to septal hypertrophy and the associated increase in extracellular space or fibrosis [31]. A prior M2-SE diffusion-weighted study in HCM reported significantly elevated MD in areas of LV fibrosis, as detected by ECV [13], but diffusion-weighted techniques do not assess FA. Together these findings suggest that the shorter diffusion time may be advantageous in identifying alterations in the dimensions and integrity of the microstructural barriers to free diffusion. However, future studies will be needed to determine the underlying mechanism behind these changes.

Both techniques demonstrated a trend toward higher systolic E2A in hypertrophied and fibrosed myocardium. Previously STEAM has shown abnormalities of E2A in HCM compared to healthy controls with elevated diastolic E2A and decreased E2A mobility globally, particularly in areas of hypertrophy [4, 7]. This relates to sheetlets retaining a more systolic, wall-perpendicular orientation in diastole.

There was an inverse correlation between FA and ECV using both M2-SE and STEAM. FA reflects the organisation of the underlying tissue, and there is decreasing organisation with lower FA values. This reduction in FA may represent a combination of myocyte disarray, myocyte hypertrophy, increased extracellular space and also diffuse or focal fibrosis. However, current DT-CMR techniques cannot distinguish the relative contribution of these and perhaps other factors.

While DT-CMR is yet to find a formal clinical role, patient studies have shown clear changes relating to the underlying disruption of the microarchitecture and its function [4, 8, 9, 12, 26, 31–34]. The scientific utility of the method is, therefore, clear and there is a growing level of interest in investigating the clinical utility of the technique within the CMR research community. When selecting the most appropriate DT-CMR technique both the sensitivity and specificity of the technique to the underlying microstructural changes of interest and the usability of method in a clinical setting must be considered. M2-SE was possible in the majority of healthy volunteers and patients during systolic contraction. Systolic FA measured using M2-SE was the only of the tensor invariants to show significant differences between LVH + LGE + and LVH − LGE − regions. In contrast, STEAM was successfully performed in both cardiac phases for all HCM patients and is the only method to have been successfully used to assess differences in dynamic sheetlet orientation during contraction via E2A mobility.

Limitations of this comparative study are the small sample size, limited spatial resolution and the lack of age and sex-matched healthy volunteers. Whilst we show that M2-SE and STEAM can discriminate phenotypically abnormal myocardium albeit with different parameter values, we cannot claim that the remaining regions of the myocardium are “normal”. As a result, we are unable to demonstrate whether DT-CMR results in those areas of myocardium free from hypertrophy and fibrosis are similar to the results in healthy myocardium. To provide a fair comparison between sequences, we matched parameters as closely as possible between M2-SE and STEAM sequences. An alternative study design may have used parameters considered optimal for each sequence. M2-SE imaging is more amenable to free breathing acquisitions than STEAM and STEAM acquisitions can take advantage of higher *b* values. However, we would only expect a substantial increase in M2-SE success rates using a higher performance gradient system (e.g., 80mT/m maximum gradient strengths). Finally frame rejection is a subjective process, dependent on the overall quality of the data set. However, the consequent blinded independent assessment of maps is a more objective marker of data quality.

In conclusion, currently only STEAM is capable of reliable biphasic assessment of the myocardium in HCM. M2-SE has a high failure rate in diastole. STEAM is therefore the current method of choice for assessing changes in microstructure during cardiac contraction, including sheetlet reorientation and the reduction in sheetlet mobility observed in cardiomyopathies.

The globally higher MD and lower FA obtained by M2-SE reflect the shorter diffusion time of this sequence. This shorter diffusion time may also explain the ability of M2-SE to detect differences between areas of LVH and fibrosis and those without via FA. However, further studies are required to examine the relationship between DT-CMR results and underlying microstructural changes such as fibrosis.

## Electronic supplementary material

Below is the link to the electronic supplementary material.

Supplementary file1 (DOCX 27 kb)

Effect of eddy current. Phantom images acquired with the STEAM and M2-SE sequence (green) are overlaid on a reference turbo-spin echo sequence (magenta) (MP4 199 kb)

## Abbreviations

bSSFP: Balanced steady state free precession
DT-CMR: Diffusion tensor cardiac magnetic resonance
E2A: Angulation of the second eigenvector
ECV: Extracellular volume fraction
EPI: Echo planar imaging
FA: Fractional anisotropy
GRAPPA: Generalised autocalibrating partially parallel acquisition
HA: Helix angle
HAG: Helix angle gradient (wall thickness normalised)
HCM: Hypertrophic cardiomyopathy
LGE: Late gadolinium enhancement
LV: Left ventricle
LVH: Left ventricle hypertrophy
M2-SE: Second-order motion-compensated spin echo
MD: Mean diffusivity
MOLLI: Modified look locker imaging
SENSE: Sensitivity encoding
STEAM: STimulated Echo Acquisition Mode
