# Supplementary material for: Diffusion tensor cardiovascular magnetic resonance in hypertrophic cardiomyopathy: a comparison of motion-compensated spin echo and stimulated echo techniques
Source: MAGMA. 2019 Nov 22;33(3):331–42. doi: 10.1007/s10334-019-00799-3 (PMC7230046; doi:10.1007/s10334-019-00799-3)
Supplement: Supplementary file 1 — Supplementary file1 (DOCX 27 kb) [file 10334_2019_799_MOESM1_ESM.docx]

**Supplementary flow chart of methods**

**Supplementary tables**

**Supplementary table 1: A comparison of global LV DT-CMR parameters between sequences.**

| **Parameter** | **STEAM systole** | **M2-SE systole** | **p** | **STEAM diastole** | **M2-SE diastole** | **p** |
| --- | --- | --- | --- | --- | --- | --- |
| MD (x10^-3^mm^2^s^-1^) | 1.151 [0.075] | 1.74 [0.25] | 0.02 | 1.27 [0.12] | 1.57 [0.27] | 0.6 |
| FA | 0.478 [0.056] | 0.341 [0.080] | 0.02 | 0.56 [0.10] | 0.396 [0.076] | 0.1 |
| HAG (˚/%) | -0.89 [0.18] | -0.71 [0.17] | 0.05 | -0.78 [0.18] | -0.76 [0.07] | 1 |
| E2A (˚) | 66.4 [1.7] | 61.2 [5.4] | 0.05 | 52.2 [5.8] | 50 [17] | 0.3 |

Median and interquartile ranges.

**Supplementary table 2: A comparison between STEAM DT-CMR results in phenotypically diseased vs. phenotypically normal regions**

| **Parameter** | **STEAM systole** | |  | **STEAM diastole** | |  |
| --- | --- | --- | --- | --- | --- | --- |
|  | **LVH+LGE+** | **LVH-LGE-** | **p** | **LVH+LGE+** | **LVH-LGE-** | **p** |
| MD (x10^-3^mm^2^s^-1^) | 1.17 [0.20] | 1.15 [0.11] | 0.43 | 1.21 [0.14] | 1.31 [0.14] | 0.19 |
| FA | 0.450 [0.092] | 0.481 [0.078] | 0.08 | 0.53 [0.12] | 0.579 [0.044] | 0.05 |
| E2A (˚) | 67.4 [6.6] | 63.2 [6.9] | 0.08 | 60 [15] | 37 [21] | 0.002 |
|  | **LVH+LGE+** | |  | **LVH-LGE-** | | **p** |
| ∆E2A (˚) | 6 [17] | |  | 21.9 [5.5] | | 0.002 |

Median and interquartile ranges. LVH – left ventricular hypertrophy, LGE – late gadolinium enhancement

**Supplementary table 3: A comparison between M2-SE DT-CMR results in phenotypically diseased vs. phenotypically normal regions**

| **Parameter** | **M2-SE systole** | |  | **M2-SE diastole** | |
| --- | --- | --- | --- | --- | --- |
|  | **LVH+LGE+** | **LVH-LGE-** | **p** | **LVH+LGE+** | **LVH-LGE-** |
| MD (x10^-3^mm2s^-1^) | 1.65 [0.25] | 1.86 [0.33] | 0.1 | 1.52 [0.31] | 1.66 [0.31] |
| FA | 0.324 [0.082] | 0.349 [0.055] | 0.02 | 0.383 [0.075] | 0.43 [0.11] |
| E2A (˚) | 64 [12] | 50 [11] | 0.05 | 55 [13] | 37 [17] |
|  | **LVH+LGE+** | |  | **LVH-LGE-** | |
| ∆E2A (˚) | 5 [13] | |  | 14 [13] | |

Note that no statistical comparison was undertaken for diastole due to insufficient data points.
